# Which factors influence health services utilization in Bulgaria? Results of a cross-sectional survey

**DOI:** 10.1093/eurpub/ckae095

**Published:** 2024-06-05

**Authors:** Elka Atanasova, Svetlana Panayotova

**Affiliations:** Department of Health Economics and Management, Faculty of Public Health, Medical University—Varna, Varna, Bulgaria; Department of Health Economics and Management, Faculty of Public Health, Medical University—Varna, Varna, Bulgaria

## Abstract

**Background:**

Research on the factors influencing health care services utilization in Bulgaria does not apply a particular model to analyze these determinants. To fill this gap, we apply the Andersen’s Behavioural Model, a commonly used framework, to determine the factors that impact the utilization of health care services in our country.

**Methods:**

Data are collected in an online survey conducted in Bulgaria in 2023 among consumers. The standardized questionnaire includes questions on the utilization of health care services used by the respondent during the preceding 12 months. We apply binary logistic regressions to analyze predictors of visits to general practitioners and medical specialists, as well as hospitalizations.

**Results:**

The results of the regression analysis show that the factors of self-reported health status and the presence of a chronic disease influence the utilization of health care services except for general practitioner visits. Greater trust in general practitioners and hospitals is associated with an increased probability of undergoing examinations and hospitalizations. Predisposing and enabling characteristics appear as significant determinants of health care utilization.

**Conclusion:**

The study confirms the relevance of the Behavioural Model for the use of health care services in the Bulgarian context. Further research on health outcomes and their impact on utilization can help determine the most efficient level and appropriateness of the use of health care services.

## Introduction

Health care utilization has always been a major concern in health policy. Recognizing the determinants of the use of health care services is essential for health policy makers to efficiently manage health care utilization and related costs. These studies can form the foundation for policies and initiatives promoting appropriate use, reducing inappropriate and excessive use of medical care and enhancing cost-effectiveness and financial stability in the health sector.

Based on the literature review with regard to factors influencing health care services utilization, we find that Andersen’s Behavioural Model of Health Services Use is the most commonly employed model, and therefore, it was chosen for the current study.^1–3^ According to this model, individuals’ use of health care services is a function of their predisposition to use such services (predisposing characteristics), of factors that enable or impede the use of services (enabling resources) and of their need for health services (need characteristics).^4^ The *predisposing* factors category includes demographic factors (e.g. age, gender), socio-structural factors (e.g. employment, educational level, place of residence) and health beliefs (people’s attitudes, values and knowledge about health and health care services). The *enabling* factors component encompasses both resources specific to individuals (e.g. income, insurance coverage, regular source of care) and also resources specific to the community in which they live (e.g. availability of physicians, hospital beds). The *need* factors represent perceived or evaluated need for care, i.e. they may be based on individuals’ own perceptions or on the diagnostic judgement of health care providers.

Overall, there are Bulgarian studies that investigated factors influencing the use of health care services.^5–7^ However, there is no reported study examining the determinants of health care services utilization using the Behavioural Model of Health Services Use by Ronald Andersen.

The health system in Bulgaria is highly centralized. The Ministry of Health is responsible for the overall organization and functioning of the health system. A compulsory social health insurance (SHI) is administered by a single payer—the National Health Insurance Fund (NHIF). In 2020, Bulgaria spent 8.5% of GDP on health, where public sources represent 65% of spending. The majority of private spending comes from out-of-pocket payments (34%), primarily for pharmaceuticals and direct payments for services not included in the NHIF benefits package.^8^ Voluntary health insurance (VHI) is provided by for-profit insurance companies, and it has a small share, accounting for about 1.3% of health care financing in Bulgaria. About 89% of the population are insured by NHIF, while the remaining 11% have to pay out-of-pocket for the health services they use.^9^ Contributions to NHIF are income-related and independent of health status.

Patients insured by NHIF have guaranteed access to a general practitioner (GP), and they can visit medical specialists with a referral. If patients visit a specialist without a GP’s referral, they are expected to pay for the service (out-of-pocket by patient for examination). Patients have different reasons and motivations for such behaviour: no SHI, shorter waiting time, use of health care services on their own decision, referral limits set by the NHIF, etc. Hospital care can be used when the GP or the specialists refer the patient to a hospital or in an emergency case. Moreover, patients have to make co-payments of BGN 2.90 (€1.50) per outpatient visit and BGN 5.80 (€2.96) for each day of hospitalization for up to 10 days per year.^10^ Although there is no overall shortage of physicians and hospitals in Bulgaria, the current distribution of human resources in the health system is unbalanced. There is a lack of GPs, and nurses are in short supply, which further erodes primary care in the country. Thus, there are significant regional distortions as well as insufficient coverage and vacancies in some specialties. The main problem in the organization of medical care in the country is the dominance of the hospital sector as a place for solving most of the population’s health problems.

The aim of the study is to identify the factors that best predict health care use in outpatient and inpatient care in Bulgaria based on Andersen’s Behavioural Model of Health Services Use and to contribute to the effort of improving health care service planning and health sector efficiency. The application of the model in our country will enable comparative analyses based on results in different countries and health systems.

## Methods

### Survey

A cross-sectional survey was carried out in January–February 2023 in Bulgaria, resulting in 1292 completed questionnaires. The survey collected data, using a standardized online questionnaire, which was sent through email and social networks. Participants are selected through the snowball sampling method. The survey collected data on individual characteristics and utilization of health care services by the respondents during the preceding 12 months.

### Outcome: health care use

Two types of services are investigated: outpatient visits to physicians and inpatient hospital services. Outpatient visits to physicians are defined as visits to GPs and medical specialists under the health insurance system, as well as outpatient visits to medical specialists in the private sector. Inpatient services are defined as hospitalizations (planned and emergency stays). The respondents are asked to state whether they have utilized the relevant health care service during the past year. The responses are structured on a dichotomous scale (visit; hospitalization: yes/no).

### Independent variables

*Predisposing factors* are included as follows: age, gender, family status (never married; married or living with a partner without marriage; divorced or widowed), place of residence (capital Sofia; regional city; small town; rural area), employment status (full-time job; part-time job or unemployed person; retired due to age or disability) and educational level [International Standard Classification of Education (ISCED 2011)].^11^ In order to analyze the health beliefs of respondents, the extent of patients’ trust in health care providers was assessed using a five-point Likert scale from 1 (“not trusted at all”) to 5 (“very much trusted”). Some obstacles in utilizing health care services are examined in an effort to better understand the health beliefs of respondents: wanted to wait and see if the problem got better on its own; being afraid of a doctor, hospital, examination or treatment; fear of COVID-19 infection; and did not know any good doctor. Respondents rate these barriers on a five-point Likert scale ranging from 1 (“this doesn’t concern me at all”) to 5 (“totally applies to me”).

*Enabling factors* are included as SHI status, VHI status and monthly household income in BGN. Additionally, some obstacles that represented enabling factors are pointed out as too expensive health care services, long waiting times or waiting lists, no time because of work and personal commitments, distance to travel and a lack of equipment and supplies at the doctor’s office. The evaluation scale is from 1 (“this does not concern me at all”) to 5 (“totally applies to me”).

*Need factors* covered self-declared health status and the presence of chronic disease. Perceived health is assessed based on a self-declared scale of 1 (“very bad”) to 5 (“very good”). The presence of chronic disease is assessed on a dichotomous scale (yes or no).

### Statistical analysis

We examine the extent to which a set of explanatory variables (predisposing, enabling and need factors/characteristics) are associated with reported utilization of health care services. Descriptive statistics are used for the presentation of the studied sample. Then, we carry out binary logistic regression analysis to identify the variables that influence the likelihood of utilization and to predict the use of health care services. This analysis was chosen because of the type of variables: one dichotomous dependent variable (use of health care service: yes/no) and multiple independent variables, represented by nominal, ordinal and interval data. The data and categories of some of the variables are transformed for regression analysis. Four logistic regressions are constructed to analyze how individual characteristics (predisposing, enabling and need characteristics) influence the likelihood of using each type of health services: GP visits, specialist visits with referrals, specialist visits without referrals (private visits) and hospital treatment.

The analyses identified the variables that have a significant and independent impact on utilization and can predict the probability of using a health care service. The findings are based on the odds ratio (OR) values, which represent the likelihood of the dependent variable falling into one of two categories (used or not used) when the independent variable’s value is compared to the reference value or increased by one unit. Statistical significance is determined at *P* < 0.05.

### Ethical issues

The collection and use of personal information before, during and after the completion of the research is carried out in accordance with the General Data Protection Regulation (Regulation 2016/679).^12^

The Research Ethics Committee of the Medical University of Varna approved the study.

## Results

### Sample characteristics

#### Predisposing characteristics

**Figure 1:**
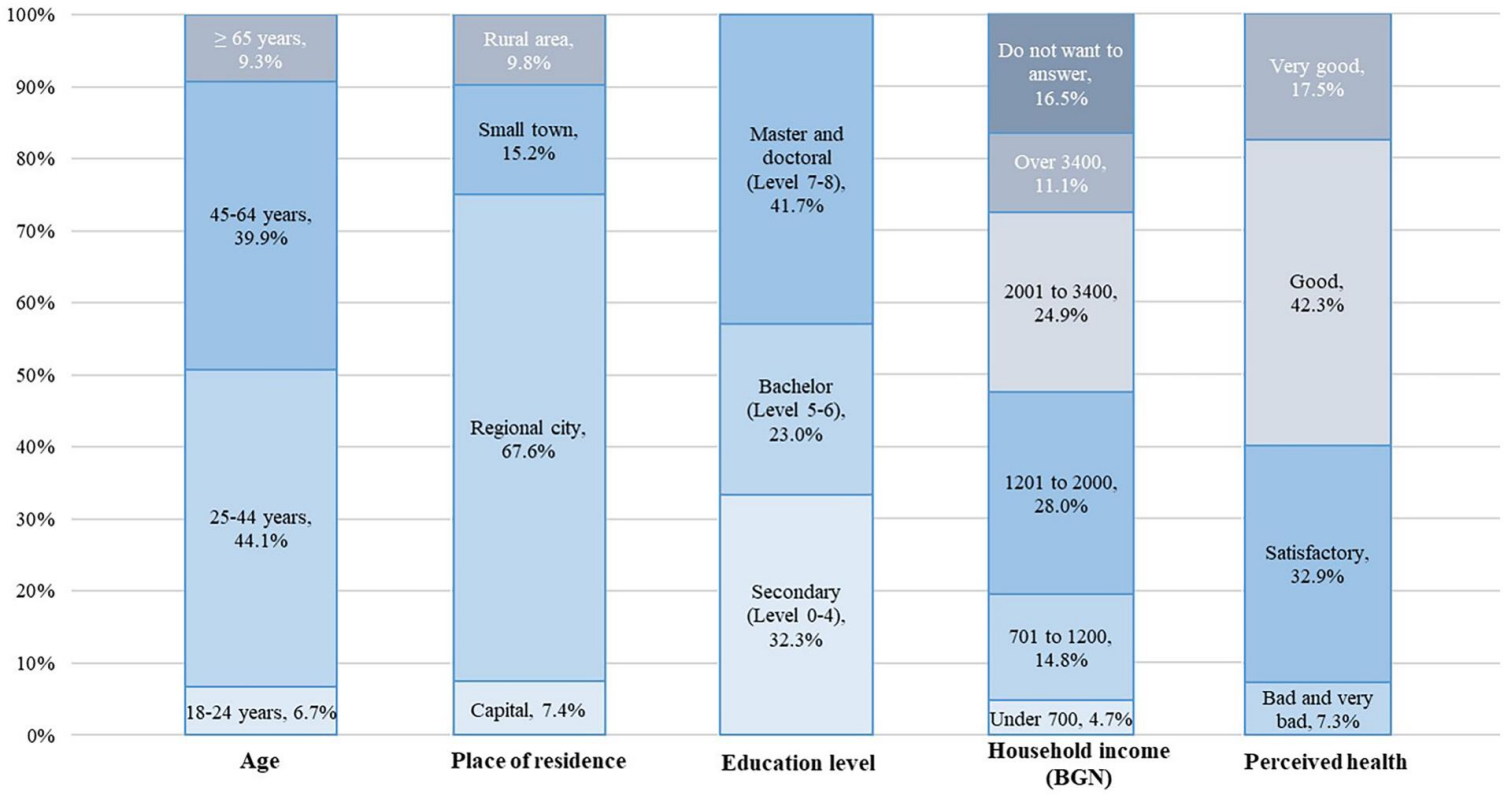
Selected predisposing, enabling, and need characteristics of the study sample (*N* = 1292).

Descriptive statistics on selected predisposing, enabling and need characteristics of the study sample are presented in figure 1. Most participants are between the ages of 25 and 44, predominantly female (77.3%), married (53.3%), residing in a regional city (67.6%), holding a master’s degree (41.7%) and employed full-time (67.0%).

The findings regarding health beliefs indicate that 82.3% of respondents trust (largely and rather) GPs, 74.9% trust medical specialists and 50.7% have trust in hospitals. Specialists have the highest average trust score of 4.11, followed by GPs at 3.92 and hospitals at 3.31 on a five-point scale. With regard to obstacles relating to health attitudes and beliefs, 33% of respondents state that they didn’t know any good doctor, and about 30% acknowledge delaying medical care until the health problem resolved itself. Twenty percent of respondents claimed that fear of COVID-19 made it difficult to get health care services.

#### Enabling characteristics

Most respondents have a household income ranging from BGN 1201–2000 (28%) and BGN 2001–3400 (24.9%). The majority of individuals (94.2%) reported that they had been continuously covered by SHI for the previous 12 months, and 21.8% stated that they had either self-funded or employer-funded VHI coverage. Respondents who have experienced moderate to significant inconvenience due to service waiting times represented 43% of the sample, while 38.4% have no sufficient time for health care services due to work and personal obligations.

#### Need characteristics

The majority of the consumers assessed their health as good—42.3%, or satisfactory—32.9%. The respondents who have one or more diagnosed chronic diseases amount to 43.7%. Cardiovascular diseases were the most frequently reported condition, accounting for 18.3% of all respondents and 41.9% of those with chronic conditions.

#### Use of health services

**Figure 2:**
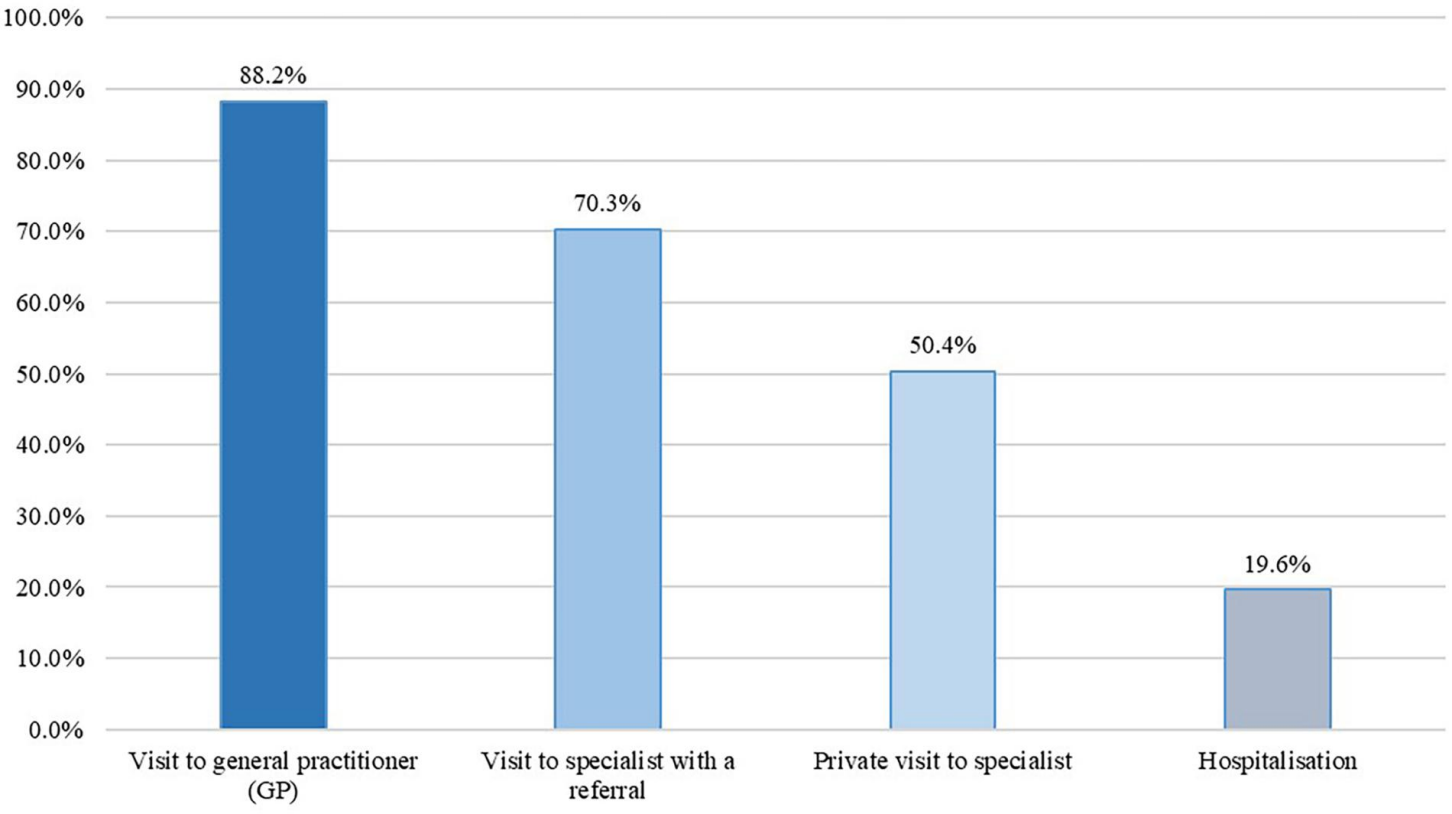
Use of health services in the last 12 months.

Of the participants, 88.2% (*N* = 1139) have visited the GP in the preceding 12 months. More than 70% of respondents (*N* = 908) were referred to a specialist for treatment and 50.4% (*N* = 651) of users of physician services undertake private visits (figure 2). The following are the main reasons to choose private visits: the fact that the preferred doctor or health facility is not contracted with the NHIF (44.2% of participants), shorter waiting times (23.8%) and demand for more than one physician’s opinion (23.5%). The average probability of hospitalizations is about 20% in the preceding 12 months.

### Main regression analysis

Table 1 presents the results of the logistic regression analysis for GP visits, specialist visits with referral, specialist visits without referral (private visits) and hospitalizations.

**Table 1:** Results from binary logistic regression analyses concerning individual characteristics associated with the use of health care services.

Individual characteristics	Visit to general practitioner (GP)		Visit to specialist with a referral		Private visit to specialist		Hospitalization	
	OR	95% CI	OR	95% CI	OR	95% CI	OR	95% CI
**Predisposing characteristics**								
Age (in years)	1.00	0.97–1.03	1.01	1.00–1.03	0.99	0.97–1.00	0.98	0.97–1.00
Gender—Female (Ref: Male)	1.37	0.66–2.86	1.52*	1.01–2.28	1.97*	1.38–2.82	1.11	0.73–1.70
Family status Never married (Ref.)								
Married/living with a partner	1.05	0.37–3.00	0.91	0.50–1.66	1.04	0.61–1.77	1.60	0.84–3.04
Divorced/widowed	0.75	0.18–3.06	0.70	0.32–1.54	1.21	0.62–2.37	1.28	0.58–2.85
Place of residence Capital (Sofia) (Ref.)								
Regional city	2.74	0.97–7.72	2.51*	1.38–4.58	0.79	0.45–1.39	0.86	0.46–1.62
Small town	2.08	0.59–7.35	2.71*	1.29–5.69	1.09	0.56–2.13	0.89	0.42–1.86
Rural area	4.53	0.87–23.51	1.46	0.66–3.19	0.78	0.38–1.61	0.72	0.32–1.65
Education level	1.06	0.83–1.36	1.01	0.89–1.16	1.16*	1.03–1.29	1.02	0.89–1.16
Employment status Full-time job (Ref.)								
Part-time job/unemployed	1.23	0.44–3.45	1.45	0.82–2.57	0.85	0.54–1.35	0.83	0.48–1.42
Retired (due to age or disability)	7.05	0.83–60.16	2.00	0.92–4.35	1.02	0.61–1.72	0.74	0.41–1.33
Didn’t know any good doctor	1.07	0.50–2.30	0.72	0.47–1.10	1.29	0.90–1.85	0.93	0.61–1.42
Fear of doctor/hospital/examination/treatment	1.32	0.54–3.23	1.02	0.62–1.70	0.64*	0.42–0.99	1.19	0.72–1.96
Fear of COVID-19 infection	0.93	0.40–2.17	0.91	0.56–1.47	0.88	0.59–1.30	0.90	0.57–1.43
Wanted to wait and see if problem got better on its own	0.57	0.28–1.15	0.75	0.51–1.11	0.95	0.68–1.34	0.68	0.45–1.03
Trust in provider	1.83*	1.43–2.34	1.04	0.86–1.28	0.94	0.79–1.11	1.31*	1.13–1.51
**Enabling characteristics**								
Household income	0.83	0.59–1.16	0.92	0.76–1.11	1.31*	1.12–1.54	0.86	0.72–1.03
SHI—Yes, continuous (Ref.: No/with interruptions)	2.16	0.66–7.09	3.52*	1.72–7.22	0.30*	0.14–0.64	1.64	0.68–3.99
VHI—Yes (Ref.: No)	1.24	0.59–2.57	0.79	0.54–1.15	1.22	0.87–1.72	0.74	0.49–1.11
Too expensive health service	0.89	0.40–2.00	1.68*	1.05–2.70	1.28	0.87–1.86	0.99	0.64–1.51
Long waiting times/waiting list	1.21	0.61–2.42	0.81	0.56–1.17	1.41*	1.03–1.93	1.25	0.87–1.81
No time (work and personal commitments)	1.19	0.60–2.35	1.00	0.69–1.46	0.90	0.65–1.25	0.63*	0.43–0.93
Distance and/or travel difficulties	0.54	0.22–1.35	0.81	0.48–1.36	1.39	0.90–2.14	1.18	0.73–1.90
Lack of equipment and supplies at the medical facility	1.23	0.50–3.03	0.87	0.53–1.43	1.29	0.84–1.98	1.12	0.69–1.82
**Need characteristics**								
Perceived health	0.66	0.37–1.18	0.66*	0.48–0.89	0.71*	0.56–0.91	0.47*	0.36–0.62
Chronic disease—Yes (Ref.: No)	1.63	0.78–3.41	1.81*	1.23–2.67	0.99	0.72–1.37	1.48*	1.01–2.16
Nadelkerkes R^2^	0.16	0.16	0.14	0.13				

Notes. OR—Odds ratio.

CI—Confidence interval.

Nadelkerkes R^2^—proportion of the dependent variable variance, explained by the full regression models, including all three components of individual factors (predisposing, enabling, and need characteristics).

* *P* < 0.05.

We find that higher trust in the provider is associated with a higher probability of visiting a GP. Additional predisposing and enabling factors do not appear to have a significant impact on consultations with the GP.

Females, residents of regional cities or small towns, individuals with continuous mandatory health insurance, those facing financial problems and chronically ill individuals are more likely to consult a specialist with a referral, while people with good self-reported health indicate a reduced probability of using this service.

We observe that being a user, women, those with higher levels of education, those with higher household income and people who faced long waiting times increased the probability of reporting visits to a specialist without a referral. Individuals with social health insurance, those who are afraid of doctors or hospitals, as well as those who are in better health are less likely to use private visits.

Higher patient trust in hospitals and the presence of a chronic disease increased the probability of hospitalization. In contrast, respondents who have no time for medical care due to personal commitments and those with good self-reported health indicate a reduced likelihood of being admitted to the hospital.

## Discussion

We find that beyond the need for health care, other factors also play an important role in the utilization of health care services. Predisposing and enabling characteristics were confirmed as independent predictors of health care use, although some factors, such as age, family status, employment status and voluntary health insurance, which previous research identified as predictors were not confirmed. We compared our results with the findings of other international studies and systematic reviews exploring Andersen’s Behavioural Model.

### Predisposing factors

Although studies often confirm a positive relationship between respondents’ age and health care service utilization,^1^^,^^2^ we do not find a strong association between age and use of health care services. Even for age, a seemingly predictable determinant of health service utilization, findings in a number of studies show inconsistencies in the strength and direction of this association.^13–17^ With respect to the use of outpatient services (specialist visits with referral and specialist visits without a referral), we observe that these visits are more frequent among females. The data from the European health interview survey carried out in the country in 2019 show a higher proportion of women who used health services (GP and specialist consultation) in the last 12 months.^18^ A lot of studies suggest an association between gender and utilization and have pronounced that women are more likely to visit a doctor.^1–3^

According to the place of residence, we find that residents of a regional city and a small town are more likely to use outpatient physician services with a referral than residents of the capital city. Our results correspond with another study in the country, which found that average utilization of GP, specialist and hospital services was higher in small towns.^19^ The systematic reviews do not provide a clear-cut relationship between place of residence and health service utilization.^1–3^

Furthermore, we observe that a higher level of education determines a higher likelihood of using private health care services, but it is not predictive of the use of other services. Findings about associations between education and health care use are ambiguous. There are international studies that report associations in either direction,^1^^,^^2^ while others find no association.^3^

Our results suggested that employment status is not a predictive factor of health care use. The influence of this factor is not often studied, and the findings are controversial. Some studies show that retirement or leaving one’s job for other reasons increased the likelihood of using specialist health services and increased GP visits,^17^ in contrast to the results of other studies, which show that regular income per household increased the utilization of outpatient health services.^2^

The relationship between trust in the provider and health service utilization is unambiguous in most cases. Higher levels of trust are associated with higher utilization, and distrust in health care institutions and medical professionals contributes to delays in seeking care and underutilization of preventive services and treatment.^13^^,^^20^ Our study similarly finds that trust in GPs and hospitals increased the probability of GP visits and hospitalizations, and people who are afraid of doctors or hospitals are less likely to use health care services without a referral. Other studies have found that distrust in health care providers increased the utilization of other types of health services.^21^^,^^22^ Lack of trust can also lead to overutilization of health care services and patients who do not trust their doctors may seek multiple additional opinions and consultations for the same diagnosis and use more expensive interventions that are not always recommended.^23^ Thus, mistrust in health care providers leads to underutilization or overutilization of health care services; in either case, the result is adverse health outcomes for patients and ineffective health care. Although our study period covers the last two months of COVID-19 in the country, fear of contracting the virus did not show a statistically significant influence on health service utilization. According to an international study, the fear of contagion is one of the main reasons for foregoing physician services.^24^

### Enabling factors

We do not observe statistical significance for GP visits, specialist visits with referrals and hospitalizations due to household income, except for paid check-ups, which suggests that higher income raises the possibility of elective services and additional consultations.

The findings of other studies investigating the association between income and health care services utilization in Bulgaria are contradictory.^7^^,^^25^ While these results do not definitively prove the existence of socioeconomic disparities in health service use in Bulgaria, it is important to note that around 19% of households in Bulgaria experienced catastrophic health expenditures in 2018, with the majority of such spending occurring in the poorest households.^8^

Social health insurance status determined a higher likelihood of visiting a specialist with a referral and a lower likelihood of using a paid examination, which reflect the organization of the health insurance model in our country. Our expectations that voluntary health insurance would be associated with a higher probability of using physician services without a referral have not been confirmed, and this factor does not emerge as significant for influencing health service utilization. Our results show that respondents who faced long wait times and waiting lists have a higher probability of using paid visits. This can be explained by people’s willingness to pay for services in order to obtain them sooner or to use them even in the event that referrals are insufficient.

### Need factors

In our study, the need factors “self-reported health” and “presence of chronic disease” influence the use of all services except GP visits, which makes sense considering the compulsory annual GP check-up. The confirmed association between health status and health service utilization suggests that needs are a leading factor and that individuals use health care services appropriately, i.e. when medically indicated, which is one of the fundamental requirements for equitable access to health care. This association has also been reported in previous international studies, which is encouraging for health care equity.^3^^,^^26^^,^^27^ We assume that utilization alone is not a reliable indicator of equity in health care, as it does not account for unmet needs.

### Strengths and limitations

This is the first study in Bulgaria which investigates the predictors of health care use and explores the framework of Andersen’s Behavioural Model. The study applies one of the most widely used models for studying the determinants of health service consumption, and this allows for comparative analyses with a large number of empirical studies conducted in different countries. Our study is not without limitations. These are mostly related to the short time period covered (a 12 month-recall period), and the cross-sectional design of the survey, with a low number of respondents in some categories. Thus, possible interactions between factors influencing health service utilization were not explored.

## Conclusions

Although there are various Bulgarian studies that investigate the utilization of health care services, we explore for the first time this issue based on Andersen’s Behavioural Model. Our results show that this model is also applicable to our country and enables comparative analyses due to its wide application by researchers. In order to achieve greater credibility of the results, it is also necessary to conduct a nationally representative survey. Moreover, further studies may go beyond the examination and description of individual factors and explore the relationships between the determinants as well as the feedback loops in the model’s structure. It is essential to discuss the extent to which each determinant can be altered to influence behaviour and utilization of health care services. Therefore, policy decisions can consider the potential of any determinant that influences utilization, in order to optimize the utilization of health services and ensure equitable access. Also, health outcomes research can help determine the most effective level and appropriateness of service utilization.

## Funding 

No funding source.

*Conflicts of interest*: None declared.

## Data Availability

The data underlying this article cannot be shared publicly due to the privacy of the individuals that participated in the study. The data will be shared on reasonable request to the corresponding author. Key points Andersen’s Behavioural Model can be applied to identify the factors influencing health services utilization in Bulgaria. Predisposing, enabling and need characteristics of the population influence the utilization of health services in the country, with a greater impact of need factors. Services are used by people who need them; however, utilization does not indicate unmet needs. Future research is needed on health outcomes and their impact on utilization.
